# Identification of metabolizing enzyme genes associated with xenobiotics and odorants in the predatory stink bug *Arma custos* based on transcriptome analysis

**DOI:** 10.1016/j.heliyon.2023.e18657

**Published:** 2023-07-27

**Authors:** Wenhong Li, Jingmiao Zou, Xiang Yang, Mingwei Yang, Po Jiang, Xinyi Wang, Chunyang Huang, Yueping He

**Affiliations:** a Institute of Plant Protection, Guizhou Academy of Agricultural Sciences, Guiyang, 550006, China; b Hubei Insect Resources Utilization and Sustainable Pest Management Key Laboratory, College of Plant Science and Technology, Huazhong Agricultural University, Wuhan, 430070, China; c Guizhou Provincial Tobacco Company Zunyi Branch, Zunyi, 563000, China

**Keywords:** *Arma custos*, Xenobiotic detoxification enzymes, Odorant degrading enzymes, Cytochrome P450 monooxygenases, Carboxylesterase, Glutathione *S*-Transferase

## Abstract

The predatory stink bug, *Arma custos*, is a highly effective beneficial predator of crop pests. The lack of gene information related to xenobiotic detoxification and odorant degrading enzymes in the predator stink bugs to date has limited our ability for more in-depth studies of biological control. Hence, we conducted *de novo* assembly of the *A. custos* transcriptome from guts, antennae, and other tiussue samples of 5th instar larvae using Illumina sequencing technology. A total of 91, 50 and 23 genes of cytochrome P450 monooxygenases (CYPs), carboxyl/choline esterases (CCEs) and glutathione *S*-transferases (GSTs) genes were identified, respectively. Gene expansions of CYP3 and CYP4 clans and the hormone and pheromone processing CCE class were found in *A. custos*. Analysis of tissue-specific expression patterns showed that 37 CYPs, 14 CCEs and 8 GSTs were enriched in guts, and 6 CYPs, 5 CCEs and 2 GSTs were up-regulated in antennae, suggesting their potential roles on xenobiotics detoxification and ordorant degradation. Gene information data presented here could be useful for a deeper understanding of the ecology, physiology and behavior of this beneficial species and could be helpful to improve their bio-control efficiency.

## 1 Introduction

*Arma custos* Fallou (Hemiptera: Pentatomidae) is a predaceous insect species that is found throughout China, Mongolia, the Korean peninsula, and other Eastern Asian regions [1,2]. It can effectively control a wide range of agricultural and forest insect pests, such as those in the orders Lepidoptera, Coleoptera, Hymenoptera and Hemiptera [2]. For this reason, *A. custos* has been studied as a biological control agent in integrated pest management (IPM) program. Currently in China, *A. custos* can be sucessfully massly bred by a number of nature enemy factories. For example, in Guizhou province, more than six millions of *A. custos* were produced annually, and releasing *A. custos* has been proven to be especially effective in the biological control of tobacco pests, such as *Spodoptera litura* and *Helicoverpa assultam*, and of *S. frugiperda* in corn and sorghum fields [3].

The importance of this insect for biological control has stimulated several studies into its biology and ecology, including its taxonomy, morphology, reproduction, geographical notes, olfactory system, predator-prey interaction, feeding strategies, mitochondrial genome and microRNAs [2,[4], [5], [6], [7], [8], [9], [10], [11], [12]]. However, the general knowledge about *A. custos* has important gaps. Currently releasing biological control program of this predatory stink bug is challenged by two issues. Firstly, the predatory bugs that are long-stem bred in factories often exhibit unsatisfactory performances in the establishment and control of the target pests in the fields. The olfactory system is critical for their poor adaptation to unfamiliar foods and environments. Wu et al. [6] identified chemosensory genes of *A. custos*, including odorant-binding proteins (OBPs), chemosensory proteins (CSPs) and chemosensory receptors. Odorant degrading enzymes (ODEs) could also be of major importance in olfaction dynamics, however, ODEs haven't been identified in *A. custos*. ODEs play pivotal roles in the inactive metabolism of exogenous odorants and in the recovery of sensitivity in the olfactory system to detect new odorants [13]. Until now, only one study was published with the genetic information about ODEs in a predaroty bug, *Picromerus lewisi* [14]. Further understanding the molecular mechanisms of the olfaction system including ODEs in *A. custos* could help us better answer these questions about field establishment of the predatory bugs.

What's more, biological control is usually accompanied by chemical control for practical applications in the field. These predatory stink bugs released in the wild fields are very difficult to avoid the exposure to insecticides. Therefore, the risk of insecticides on *A. custos* should not be ignored in IPM. Information of insecticide toxicities against the predators is helpful for exploiting insecticides which do not harm predators and subsequently improve the efficacy of IPM strategies. Our previous studies showed that imidacloprid, thiamethoxam and pyrethoids exhibited high toxicities against *A. custos* while sulfoxaflor, chlorantraniliprole and a couple of bio-insecticides had relatively low toxicitites against *A. custos* [3]. The genetic information about insecticide detoxification was revealed in a couple of predatory bugs, including *Cyrtorhinus lividipennis*, *P. lewisi*, and *Eocanthecona furcellata* [[14], [15], [16], [17]]. However, genetic information on *A. custos* in response to chemical insecticides is lacking. Only two recent studies had assessed differential expression profiles of the detoxification-related genes in *A. custos* under exposure to *Bacillus thuringiensis* (Bt) toxins and Transgenic Bt cotton [18,19]. In-depth investigation of mechanisms of insecticide detoxification of the predators could help us to develop successful IPM strategies based on a better balance between chemical and biological control methods.

Insects rely mainly on three superfamilies of metabolizing enzymes to degrade environmental chemicals including odorants, insecticides and other xenobiotics: cytochrome P450 monooxygenases (CYPs), carboxyl/choline esterases (CCEs) and glutathione *S*-transferases (GSTs) [20]. However, the genetic information related to these metabolizing enzymes of *A. custos* is lacking. The aim of the work reported here was to uncover the genes of these three metabolizing enzyme families in *A. custos* by analyzing the transcriptomics and expression profile data. These results provide a comprehensive foundation for further studies of molecular mechanisms of processing environmental chemicals in the predatory stink bug, which could be helpful to develop better strategies for the use of these biological control agents.

## 2 Materials and methods

### 2.1 Insects

The *A. custos* individuals used in the present study belonged to a colony reared in the natural enemy breeding center at Fenggang County of The Guizhou Provincial Tobacco Company Zunyi Branch, Zunyi, China. The bugs were reared continuously on *Mythimna separata* larvae that were fed with artificial diet mainly consisting of corn leaf powder. They were reared under 27 ± 1 °C, RH of 75 ± 5%, and a 16 h:8 h (L:D) photoperiod.

### 2.2 Sample preparation and RNA sequencing

Guts (AcG), antennae (AcA), salivary glands (AcSG), legs (AcL) and heads without antennae and salivary glands (AcH) from 100 nymphs at the fifth instar stage of *A. custos* were isolated for total RNA extraction using TRIzol reagent (Invitrogen, USA). Nymphs of *A. custos* were starved for at least 12 h prior to extraction of RNAs. Three biological replicates were perfomed on each tissue sample. In this study, RNA sequencing (RNA-seq) was carried out at Novogene Bioinformatics Technology Co., China. Using illumina NovaSeq 6000 (illumina, USA), the end reading of 150 bp pairing is generated. Each sample generated approximately 20 million sequence reads.

Raw data were firstly processed through in-house perl scripts by removing reads containing adapter or poly-N and low-quality reads. At the same time, Q20, Q30 and GC content of the clean data were calculated. The clean reads were then assembled into unigenes using the Trinity software (v2.6.6) [21].

Databases of the non-redundant protein sequences of National Center for Biotechnology Information (NCBI NR), NCBI nucleotide sequences (NT), Kyoto Encyclopedia of Genes and Genomes (KEGG), Swiss-prot, Gene Ontology (GO), euKaryotic Ortholog Groups (KOG) and Protein family (Pfam) were used to *de novo* annotate all unigenes. The reads of all libraries were deposited in the NCBI SRA (Short Read Archive) with BioProject accession number of PRJNA878928.

### 2.3 Differential expression analysis

DESeq2 R package (1.20.0) [22] were used for differential expression analysis. Expression of unigenes was calculated using FPKM (fragments per kb per million fragments) [23]. Benjamini and Hochberg's procedure was adopted to calculate the resulting *P*-values (P_adj) for controlling the false discovery rate (FDR) [24]. Differentially expressed genes (DEGs), defined as | log2 (fold) | > 1 and P_adj <0.05, were screened. Heatmaps were constructed to visualize transcriptomic data based on log10 (FPKM+1) values [25].

### 2.4 Identification and analysis of metabolizing enzyme genes

Identification of genes encoding metabolizing enzymes, CYPs, CCEs and GSTs, of *A. custos* was firstly performed via keyword searches of the *A. custos* transcriptome annotation table. The annotated CYP, CCE and GST protein sequences of three bugs, *Halyomorpha halys* [26], *Rhodnius prolixus* [27] and *Cyrtorhinus lividipennis* [15], were used for BLAST queries against the *A. custos* transcriptome to identify any missed genes. Putative metabolizing enzyme genes were in turn used as queries for additional blasting until no new candidates could be found.

Phylogenetic trees and NCBI blast searches were used to identify subfamilies of *A. custos* metabolizing enzyme genes. After manually purging small gene fragments, phylogenetic trees were constructed based on the amino acid sequence alignment of metabolizing enzyme genes from *A. custos* and other bug species by applying MEGA version 6 software [28], using the maximum likelihood method, the Jones-Taylor-Thornton (JTT) model and a bootstrap analysis with 1000 replicates. Subsequently, subfamily assignment for small gene fragments without involving in the phylogenetic trees were performed by the blast searches against NCBI databases and the annotated CYP, CCE and GST protein sequences of *H. halys* [26], *R. prolixus* [27] and *C. lividipennis* [15].

### 2.5 Quantitative real-time PCR (qRT-PCR) validation of RNA-seq data

In order to validate the RNA-seq data, fifteen genes were selected for qRT-PCR analysis using SYBR Premix Ex Taq (Takara Biotechnology Corporation Co. Ltd, Dalian, China) and an ABI Prism 7300 (Applied Biosystems, Foster City, CA). A list of qRT-PCR primers was presented in Table S1. EF1A (Cluster-13084.11009) was selected for the candidate reference genes. Regular PCR assays and melting curve anlyses were performed to verify the specificity of primers (Figure S1). The qRT-PCR assays were performed with three independent biological replicates and three technical repeats. Relative expression levels for each gene were calculated using the 2^−ΔΔCt^ method [29]. The correlation analysis of fold change (FC) data (log2 transformation) between RNA-Seq and qRT-PCR was performed using Pearson correlation analysis by SAS (version 8.01, SAS Inc., Cary, USA).

## 3 Results

### 3.1 RNA sequencing and transcript assembly

A total of 15 cDNA libraries, three each from guts (G), salivary glands (SG), antennae (A), legs (L) and heads (H), were produced from fifth-instar larvae of *A. custos*. The raw sequence (with more than 19.4 million reads in each sample) was processed for trimming and filtering, resulting in the Q20 value higher than 97% in each sample (Table S2). The transcriptomics from tissue samples resulted in a total of 41,845 unigenes with a mean length of 1219 bp, and a N50 length of 2183 bp. About 50% of 41,845 unigenes matched known proteins, after annotated with NR, NT, KEGG, Swiss-prot, GO, KOG and Pfam databases. Similarity distribution and species distribution of unigenes that hit in the NCBI NR protein database are shown in Figure S2.

### 3.2 Identification and tissue-specific expression analysis of CYPs

The insect CYP superfamily includes four clans: CYP2, CYP3, CYP4 and the mitochondrial CYP clan (CYPmito), which in turn are subdivided into families and subfamilies [30]. In the *A. custos* transcriptome, 91 putative genes or gene fragments were annotated as CYPs (Table S3). Based on phylogeny trees and the BLAST searches against NCBI databases and the named CYPs of *H. halys* [26], *R. prolixus* [27] and *C. lividipennis* [15], they were identified and sorted into four CYP clans and 27 families. Specifically, 48 genes were classified to the CYP3 clan, 31 to the CYP4 clan, 5 to the CYP2 clan and 7 to the CYPmito clan (Table 1). The number of *A. custos* CYP genes is much less than those from *H. halys*, *R. prolixus* and *Triatoma infestans*, but much more than those from *Orius laevigatus*, *C. lividipennis*, *Cimex lectularius* and *Murgantia histrionica* (Table 1). *A. custos* seems to have a larger species-specific expansion on CYP3 and CYP4 clans compared to the other two predatory bugs, *O. laevigatus* and *C. lividipennis* (Table 1).

**Table 1:** Numbers of CYP genes annotated in *A. custos* (in this study), *Orius laevigatus* [31], *Cyrtorhinus lividipennis* [15], *Rhodnius prolixus* [32], *Triatoma infestans* [32], *Halyomorpha halys* [26] and *Murgantia histrionica* [33].

Clan	Predatory bugs			Hematophagous bugs		Herbivory bugs	
	Arma custos	Orius laevigatus	Cyrtorhinus lividipennis	*Rhodnius prolixus*	*Triatoma infestans*	Halyomorpha halys	Murgantia histrionica
CYP2	5	6	5	7	1	6	7
CYP3	48	34	27	55	65	87	43
CYP4	31	13	21	49	22	45	30
CYPmito	7	5	4	8	6	6	6
Total	91	58	57	119	94	141	86

**Fig. 1:**
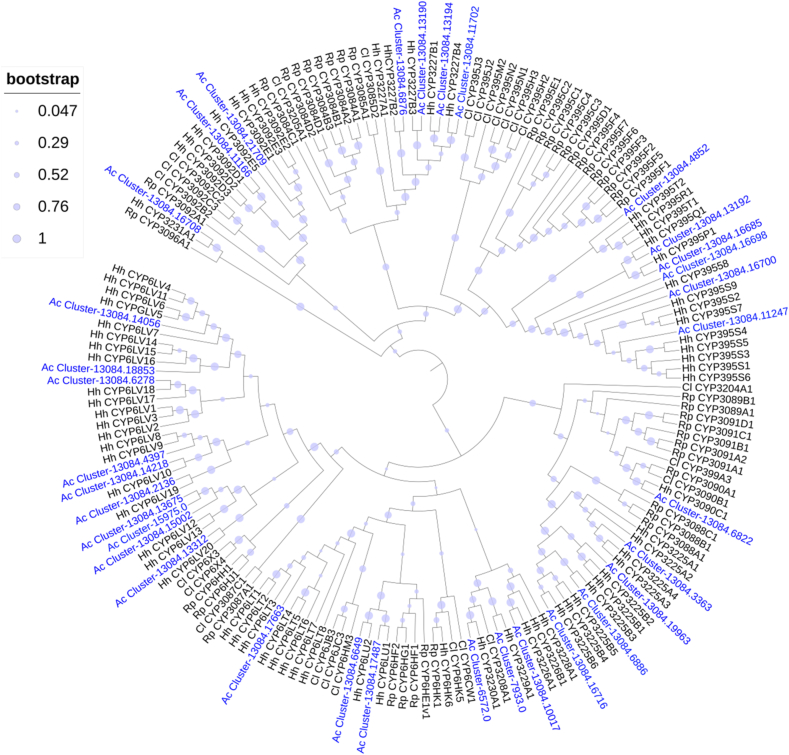
Phylogenetic tree of the CYP3 clan genes of *A. custos* (Ac), *Halyomorpha halys* (Hh) [26], *Rhodnius prolixus* (Rp) [27,32] and *Cyrtorhinus lividipennis* (Cl) [15], constructed by the Maximum likelihood method with the JTT model.

The majority of annotated CYP genes in the *A. custos* transcriptome belonged to the CYP3 clan (48 genes), which is represented by the CYP6 family (20 genes), by the CYP395 family (10 genes) and by 18 genes of new 9 families (CYP3085, CYP3088, CYP3090, CYP3092, CYP3096, CYP3208, CYP3225, CYP3226, CYP3227) (Table 2). Same as other Hemiptera species, *A. custos* has no genes belonging to the CYP9 family. In the maximum likelihood tree of CYP3 clan, 34 full or nearly full-length sequences of CYP3 genes in *A. custos* were involved (Fig. 1).

**Table 2:** FPKM values of CYP3 clan genes in different tissues/parts of *A. custos*.

Clan	Family	Gene ID	Log10(FPKM+1)			Log2(Fold)	
			G	A	H	G/H	A/H
CYP3	CYP3085	Cluster-13084.11702	2.57	0.85	1.36	4.07*	−1.83
CYP3	CYP395	Cluster-13084.11247	2.43	1.18	2.10	1.09*	−3.15
CYP3	CYP3088	Cluster-13084.6886	2.32	0.43	1.45	2.93*	−4.00
CYP3	CYP3226	Cluster-13084.10019	1.14	2.22	1.70	−1.97	1.74*
CYP3	CYP3088	Cluster-13084.16716	2.14	0.11	1.54	2.02*	−6.80
CYP3	CYP6	Cluster-13084.17487	2.05	0.05	0.51	5.66*	−4.09
CYP3	CYP6	Cluster-13084.6649	2.04	0.29	0.83	4.26*	−2.55
CYP3	CYP395	Cluster-13084.6141	1.99	0.10	1.25	2.53*	−6.05
CYP3	CYP395	Cluster-13084.16700	1.93	0.36	1.41	1.75*	−4.27
CYP3	CYP6	Cluster-13084.17663	1.92	0.00	0.13	7.91*	−5.09
CYP3	CYP3088	Cluster-13084.3363	0.42	0.23	1.91	−5.62	−6.89
CYP3	CYP3090	Cluster-13084.6822	1.88	0.06	0.46	5.30*	−3.67
CYP3	CYP395	Cluster-13084.13192	1.84	0.48	0.98	3.02*	−2.07
CYP3	CYP395	Cluster-13084.4852	1.82	0.80	1.20	2.15*	−1.48
CYP3	CYP3225	Cluster-13084.6890	1.82	0.12	0.89	3.28*	−4.40
CYP3	CYP3092	Cluster-13084.11166	1.66	1.20	0.72	3.37*	1.79*
CYP3	CYP6	Cluster-13084.13675	1.63	0.86	0.96	2.36*	−0.37
CYP3	CYP395	Cluster-13084.16685	1.63	0.10	0.73	3.24*	−4.14
CYP3	CYP6	Cluster-13084.7361	1.61	0.27	0.80	2.89*	−2.64
CYP3	CYP6	Cluster-13084.14218	0.97	0.92	1.48	−1.83	−2.02
CYP3	CYP6	Cluster-13084.6278	1.48	0.00	0.08	7.18*	−4.32
CYP3	CYP6	Cluster-13084.15002	1.40	0.51	1.02	1.33*	−2.08
CYP3	CYP6	Cluster-13084.4397	1.38	0.01	0.11	6.34*	−3.22
CYP3	CYP3092	Cluster-13084.21709	0.27	0.00	1.16	−3.98	–
CYP3	CYP3085	Cluster-13084.13190	1.28	0.17	0.32	4.06*	−1.18
CYP3	CYP3085	Cluster-13084.13194	1.24	0.40	0.67	2.16*	−1.28
CYP3	CYP6	Cluster-13084.14056	1.24	0.29	0.70	2.00*	−2.10
CYP3	CYP395	Cluster-13084.10017	0.24	1.23	0.83	−2.99	1.49*
CYP3	CYP6	Cluster-13084.13312	1.23	0.73	1.09	0.50	−1.36
CYP3	CYP395	Cluster-13084.6564	1.14	0.23	0.87	0.99	−3.25
CYP3	CYP6	Cluster-13084.15822	1.03	0.96	0.89	0.52	0.28
CYP3	CYP6	Cluster-13084.18853	1.02	0.12	0.22	3.88*	−0.98
CYP3	CYP3088	Cluster-13084.19963	0.19	0.14	1.02	−4.08	−4.60
CYP3	CYP3096	Cluster-13084.16708	0.98	0.24	0.53	1.87*	−1.65
CYP3	CYP6	Cluster-13084.12669	0.08	0.94	0.86	−5.05	0.31
CYP3	CYP6	Cluster-13084.13186	0.94	0.24	0.74	0.77	−2.61
CYP3	CYP3085	Cluster-13084.6876	0.28	0.02	0.70	−2.13	−6.31
CYP3	CYP6	Cluster-13084.2136	0.09	0.01	0.69	−4.15	−7.02
CYP3	CYP3208	Cluster-6572.0	0.64	0.05	0.01	6.82*	2.12*
CYP3	CYP395	Cluster-13084.13191	0.59	0.32	0.62	−0.13	−1.55
CYP3	CYP6	Cluster-7933.0	0.61	0.02	0.11	3.45*	−2.49
CYP3	CYP6	Cluster-13084.777	0.24	0.33	0.53	−1.71	−1.05
CYP3	CYP395	Cluster-13084.16698	0.52	0.03	0.15	2.54*	−2.51
CYP3	CYP3231	Cluster-13084.15016	0.37	0.06	0.17	1.52*	−1.75
CYP3	CYP6	Cluster-15975.0	0.08	0.13	0.26	−1.93	−1.23
CYP3	CYP3227	Cluster-13084.15845	0.23	0.04	0.07	2.06*	−0.92
CYP3	CYP6	Cluster-13768.0	0.00	0.15	0.22	–	−0.67
CYP3	CYP3227	Cluster-15249.0	0.02	0.09	0.09	−2.20	−0.06

Note: * indicated that the expression level of one gene in guts (G) or antennae (A) was significantly higher than that in heads (H), with log2(fold) > 1 and P_adj<0.05. The heatmap scale was based on log10 (FPKM+1) values: Min = 0.0 Max = 2.57.

**Fig. 2:**
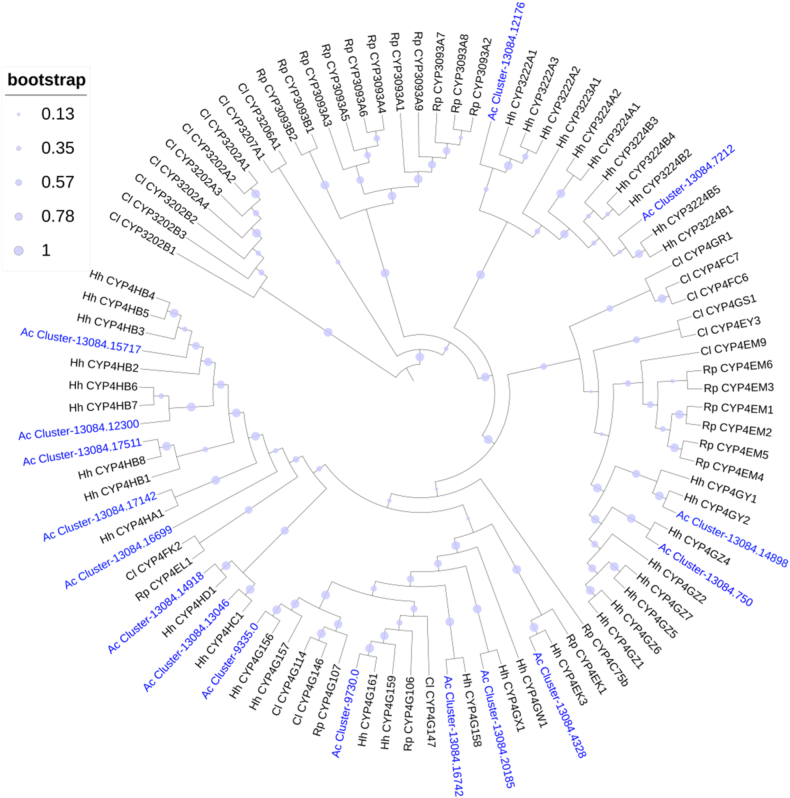
Phylogenetic tree of the CYP4 clan genes of *A. custos* (Ac), *Halyomorpha halys* (Hh) [26], *Rhodnius prolixus* (Rp) [27,32] and *Cyrtorhinus lividipennis* (Cl) [15], constructed by the Maximum likelihood method with the JTT model.

The CYP4 clan contains 31 genes belonging to the CYP4 family (19 genes) and to the new families (CYP3222, CYP3223, and CYP3224) (Table 3). In the maximum likelihood tree of CYP4 clan, 16 full or nearly full-length sequences of CYP4 genes in *A. custos* were involved (Fig. 2).

**Table 3:** FPKM values of CYP4 clan genes in different tissues/parts of *A. custos*.

Clan	Family	Gene ID	Log10(FPKM+1)			Log2(Fold)	
			G	A	H	G/H	A/H
CYP4	CYP4	Cluster-13084.17142	2.47	0.08	1.12	4.60*	−5.94
CYP4	CYP4	Cluster-13084.16699	1.83	0.15	0.70	4.06*	−3.28
CYP4	CYP4	Cluster-13084.5960	1.73	0.27	0.12	7.31*	1.42*
CYP4	CYP4	Cluster-13084.14898	1.63	0.03	1.27	1.24*	−7.97
CYP4	CYP4	Cluster-13084.15717	1.58	0.97	1.43	0.51	−1.61
CYP4	CYP4	Cluster-13084.12300	0.84	1.44	1.18	−1.24	0.89
CYP4	CYP4	Cluster-13084.17511	1.28	0.17	0.68	2.25*	−3.02
CYP4	CYP3224	Cluster-13084.7214	0.31	1.27	1.11	−3.51	0.55
CYP4	CYP4	Cluster-13084.13046	0.77	0.85	1.16	−1.48	−1.14
CYP4	CYP4	Cluster-13084.20185	0.11	1.09	0.87	−4.47	0.82
CYP4	CYP4	Cluster-13084.16742	0.85	0.06	0.91	−0.22	−5.57
CYP4	CYP4	Cluster-13084.14918	0.48	0.71	0.87	−1.69	−0.63
CYP4	CYP3224	Cluster-13084.7212	0.25	0.73	0.64	−2.12	0.37
CYP4	CYP3222	Cluster-13084.12176	0.17	0.44	0.72	−3.14	−1.28
CYP4	CYP3224	Cluster-13084.7215	0.12	0.62	0.60	−3.22	0.07
CYP4	CYP4	Cluster-9335.0	0.61	0.03	0.15	2.92*	−2.36
CYP4	CYP3224	Cluster-13084.7213	0.00	0.20	0.52	–	−2.03
CYP4	CYP3224	Cluster-13084.7211	0.04	0.52	0.29	−3.37	1.31*
CYP4	CYP4	Cluster-9730.0	0.44	0.06	0.15	2.08*	−1.58
CYP4	CYP4	Cluster-681.0	0.00	0.00	0.00	–	–
CYP4	CYP3224	Cluster-13084.1619	0.00	0.09	0.39	–	−2.69
CYP4	CYP4	Cluster-13084.4328	0.01	0.02	0.05	−2.12	−1.38
CYP4	CYP3223	Cluster-10163.0	0.10	0.38	0.19	−1.03	1.36
CYP4	CYP4	Cluster-13084.750	0.10	0.31	0.36	−2.30	−0.33
CYP4	CYP3224	Cluster-13084.1620	0.00	0.03	0.32	–	−3.99
CYP4	CYP3224	Cluster-14043.0	0.00	0.23	0.32	–	−0.66
CYP4	CYP4	Cluster-13084.18399	0.00	0.25	0.27	–	−0.16
CYP4	CYP3224	Cluster-14043.1	0.00	0.00	0.28	–	–
CYP4	CYP4	Cluster-8751.0	0.23	0.05	0.08	1.81	−0.74
CYP4	CYP3224	Cluster-13084.1618	0.00	0.11	0.21	–	−1.10
CYP4	CYP4	Cluster-13084.22402	0.02	0.08	0.17	−3.55	−1.31

Note: * indicated that the expression level of one gene in guts (G) or antennae (A) was significantly higher than that in heads (H), with log2(fold) > 1 and P_adj<0.05. The heatmap scale was based on log10 (FPKM+1) values:Min = 0.0 Max = 2.47.

**Fig. 3:**
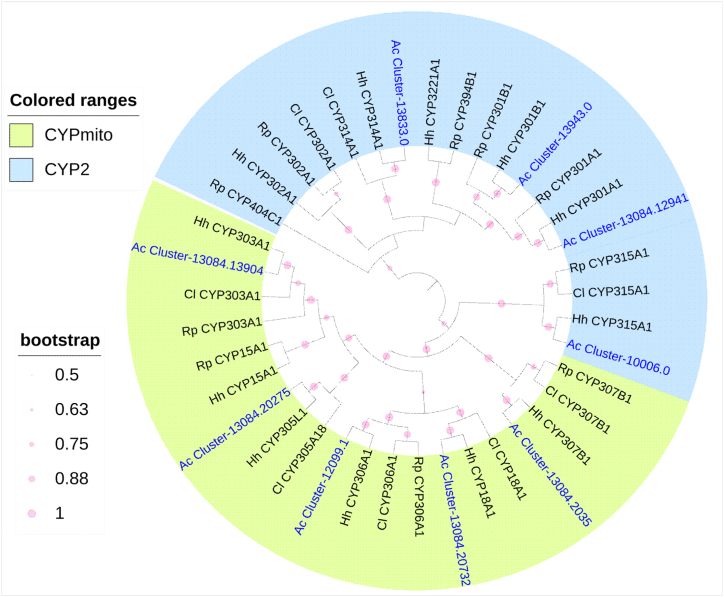
Phylogenetic tree of the CYP2 clan and CYPmito genes of *A. custos* (Ac), *Halyomorpha halys* (Hh) [26], *Rhodnius prolixus* (Rp) [27,32] and *Cyrtorhinus lividipennis* (Cl) [15], constructed by the maximum likelihood method with the JTT model.

In *A. custos*, the CYP2 clan contains one gene of each CYP18, CYP303, CYP305, CYP306, and CYP307 families (Table 4; Fig. 3). The CYP15 gene was not detected in the *A. custos* transcriptome. The mitochondrial clan contains one gene of each CYP301A1, CYP301B1, CYP314A1, CYP315A1 and CYP3221 subfamilies, and two gene fragments of the CYP302 famliy (Table 4; Fig. 3).

**Table 4:** FPKM values of CYP2 clan and CYPmito clan genes in different tissues/parts of *A. custos*.

Clan	Family	Gene ID	Log10(FPKM+1)			Log2(Fold)	
			G	A	H	G/H	A/H
CYP2	CYP303	Cluster-13084.13904	0.09	0.85	1.33	−6.41	−1.75
CYP2	CYP18	Cluster-13084.20732	0.01	0.32	1.18	−8.89	−3.69
CYP2	CYP307	Cluster-13084.2035	0.44	0.03	1.08	−2.65	−7.30
CYP2	CYP305	Cluster-13084.20275	0.62	0.29	0.83	−0.86	−2.57
CYP2	CYP306	Cluster-12099.1	0.08	0.15	0.50	−3.36	−2.43
CYPmito	CYP301	Cluster-13943.0	0.08	0.46	0.83	−4.77	−1.59
CYPmito	CYP301	Cluster-13084.12941	0.05	0.27	0.66	−4.91	−2.08
CYPmito	CYP314	Cluster-13833.0	0.06	0.30	0.58	−4.14	−1.51
CYPmito	CYP3221	Cluster-13084.19442	0.27	0.25	0.44	−1.06	−1.16
CYPmito	CYP315	Cluster-10006.0	0.39	0.10	0.42	−0.16	−2.72
CYPmito	CYP302	Cluster-6849.0	0.22	0.00	0.25	−0.24	–
CYPmito	CYP302	Cluster-2236.0	0.22	0.00	0.18	0.38	–

Note: * indicated that the expression level of one gene in guts (G) or antennae (A) was significantly higher than that in heads (H), with log2(fold) > 1 and P_adj<0.05. The heatmap scale was based on log10 (FPKM+1) values:Min = 0.0 Max = 1.33.

In order to identify candidated degrading genes responding to xenobiotics and odors, expression patterns among guts, antennae and other tissues/parts of *A. custos* nymphs were examined based on their FPKM values. To validate the DEGs identified through RNA-seq sequencing, fifteen genes were selected for qRT-PCR analysis. The results of linear regression analysis indicated a significant correlation (R^2^ = 0.70, *F* = 64.35, *P* < 0.0001) between the data of qRT-PCR and RNA-Seq (Figure S3).

In Table 2, Table 3, Table 4, FPKM values of CYPs in guts, antennae and heads were exhibited. Amongst the 48 CYP3 clan genes, 30 showed significantly higher expression levels in guts than heads, whereas 4 were significantly up-regulated in anntenna than heads, based on a criterion of Log2 (fold) > 1 and P_adj<0.05 for significant difference (Table 2). Of the 31 CYP4 genes, 7 and 2 were significantly more expressed in guts and anntenna than heads, respectively (Table 3). All of the 14 members in CYP2 and CYPmito clans exhibited predominant expression levels in heads (Table 4).

### 3.3 Identification and tissue-specific expression analysis of CCEs

**Fig. 4:**
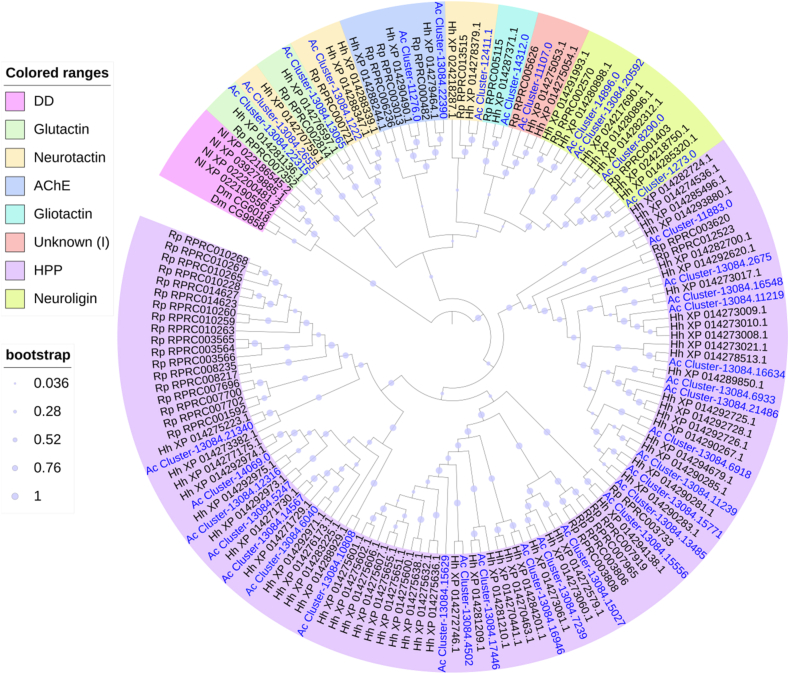
Phylogenetic tree of the CCE genes of *A. custos* (Ac), *Halyomorpha halys* (Hh) [26] and *Rhodnius prolixus* (Rp) [27,32] constructed by the Maximum likelihood method with the JTT model. Two *Drosophila melanogaster* (Dm) dietary/detoxification (DD) class genes and four *Nilaparvata lugens* (Nl) DD class genes were added into the tree, in order to showing the absent of DD class in Heteroptera. Expasion of the hormone and pheromone processing (HPP) class in bugs were shown in the tree.

Insect CCEs is typically divided into three functional classes: dietary/detoxification (DD), hormone and pheromone processing (HPP) and neuro/developmental (ND) [20,[3][4], [3][5], [3][6]]. A total of 50 putative CCE genes or gene fragments, after annotating the transcriptome and removing a couple of small redundancies (Table S3; Table 5, Table 6). Our results revealed that DD CCEs are absent in *A. custos*, which is consistent with recent studies that DD class is absent in hematophagous bugs, predatory *O. laevigatus*, and herbivory Pentatomidae bugs [31]. Numbers of *A. custos* CCE genes in the HPP and ND classes are 32 and 18, respectively (Table 5). In the maximum likelihood tree of CCEs, 39 full or nearly full-length sequences of CCEs in *A. custos* were involved (Fig. 4). The HPP class in *A. custos* includes secreted β-esterases and juvenile hormone esterases (JHEs). It shows an expansion of the HPP class in *A. custos* compared with other two predatory bugs (Table 5). The ND class in *A. custos* contains catalytic acetylcholinesterases (AchE-1 and AchE-2) and non-catalytic neuroligin, glutactin, gliotactin, neurotactin and uncharacterized clade I proteins (Table 6).

**Table 5:** Numbers of CCE genes annotated in *A. custos* (in this study), *Orius laevigatus* [31], *Cyrtorhinus lividipennis* [15], *Rhodnius prolixus* [27,32], and *Halyomorpha halys* [26]. *The number of CCE genes in *Halyomorpha halys* was revised to 76 after deleting 6 alternative splicing variants based on Sparks et al. [26].

Class	*Arma custos*	*Orius laevigatus*	*Cyrtorhinus lividipennis*	*Rhodnius prolixus*	*Halyomorpha halys*
Detoxification/Dietary (DD)	0	0	0	0	0
Pheromone/hormone (HPP)	32	16	12	40	55
Neuro/Developmental (ND) (total)	18	16	14	12	21
Clade H – Glutactin	2	1	2	2	2
Clade J – AChE	2	2	2	2	2
Clade K – Gliotactins	1	3	1	1	1
Clade L – Neuroligins	6	8	7	13	9
Clade M − Neurotactins	4	1	1	2	4
Unknown Function	3	1	1	1	3
Total COEs	50	32	26	61	76*

**Table 6:** FPKM values of CCE genes in different tissues/parts of *A. custos*.

Class	Family	Gene ID	Log10(FPKM+1)			Log2(Fold)	
			G	A	H	G/H	A/H
HPP	Beta esterases	Cluster-13084.16634	2.93	0.07	0.65	7.94*	−4.28
HPP	Beta esterases	Cluster-13084.6040	2.61	0.89	1.70	3.04*	−2.87
HPP	Beta esterases	Cluster-13084.12859	0.67	2.51	1.16	−1.88	4.61*
HPP	Beta esterases	Cluster-13084.11239	2.29	0.85	0.42	6.89*	1.88*
HPP	Beta esterases	Cluster-13084.6933	2.22	0.56	1.20	3.45*	−2.49
HPP	Beta esterases	Cluster-13084.12316	1.55	1.78	2.07	−1.75	−0.97
HPP	Beta esterases	Cluster-13084.10808	0.04	1.96	0.68	−5.23	4.60*
HPP	Beta esterases	Cluster-13084.11219	1.83	0.57	0.71	4.00*	−0.64
HPP	Beta esterases	Cluster-13084.2675	0.78	0.46	1.83	−3.72	−5.12
HPP	Beta esterases	Cluster-13084.6013	1.77	0.02	0.18	6.86*	−3.64
HPP	Beta esterases	Cluster-13084.5247	1.75	0.79	1.45	1.04*	−2.42
HPP	Beta esterases	Cluster-13084.16548	1.62	0.20	0.46	4.42*	−1.71
HPP	Beta esterases	Cluster-13084.13485	0.39	1.59	0.67	−1.34	3.38*
HPP	Beta esterases	Cluster-13084.6918	1.44	0.04	0.28	4.86*	−3.34
HPP	Beta esterases	Cluster-13084.14567	0.00	1.33	0.24	**-**	4.80*
HPP	Beta esterases	Cluster-13084.15556	0.11	1.07	1.17	−5.51	−0.35
HPP	Beta esterases	Cluster-13084.21486	1.13	0.50	0.98	0.54	−1.98
HPP	Beta esterases	Cluster-13084.21340	0.13	0.58	1.05	−4.83	−1.86
HPP	Beta esterases	Cluster-13084.15771	0.09	0.91	0.84	−4.74	0.28
HPP	Beta esterases	Cluster-11883.0	0.12	0.06	0.86	−4.30	−5.30
HPP	Beta esterases	Cluster-13084.20473	0.00	0.15	0.54	**-**	−2.60
HPP	Beta esterases	Cluster-14069.0	0.07	0.15	0.37	−2.87	−1.65
HPP	Beta esterases	Cluster-6499.0	0.24	0.00	0.05	**2.51**	–
HPP	Beta esterases	Cluster-13084.7112	0.15	0.05	0.16	−0.10	−1.79
HPP	Beta esterases	Cluster-13084.3117	0.00	0.00	0.15	**-**	–
HPP	JHE	Cluster-13084.15629	2.44	0.21	0.55	6.73*	−2.02
HPP	JHE	Cluster-13084.16946	2.26	0.71	1.01	4.29*	−1.15
HPP	JHE	Cluster-13084.17446	2.12	0.01	0.23	7.58*	−4.50
HPP	JHE	Cluster-13084.7239	2.10	0.85	1.48	2.1*	−2.25
HPP	JHE	Cluster-13084.6967	1.69	0.00	0.13	7.05*	−5.17
HPP	JHE	Cluster-13084.15027	0.35	0.69	1.01	−2.92	−1.24
HPP	JHE	Cluster-13084.4502	0.24	0.00	0.90	−3.22	–
ND	AChE	Cluster-13084.22390	0.10	0.16	0.80	−4.42	−3.54
ND	AChE	Cluster-11276.0	0.05	0.31	0.47	−4.04	−0.91
ND	Gliotactin	Cluster-14312.0	0.45	0.20	0.55	−0.47	−2.08
ND	Glutactin	Cluster-13084.13065	0.71	1.18	1.92	−4.29	−2.54
ND	Glutactin	Cluster-13084.22315	0.56	0.02	1.08	−2.06	−8.12
ND	Neuroligin	Cluster-14896.0	0.01	0.26	0.65	−7.42	−2.10
ND	Neuroligin	Cluster-13084.20592	0.13	0.02	0.63	−3.20	−6.05
ND	Neuroligin	Cluster-8290.0	0.00	0.00	0.50	−7.75	−7.75
ND	Neuroligin	Cluster-1190.0	0.00	0.00	0.26	**-**	–
ND	Neuroligin	Cluster-516.0	0.00	0.00	0.27	**-**	–
ND	Neuroligin	Cluster-1273.0	0.00	0.00	0.29	**-**	–
ND	Neurotactin	Cluster-13084.2655	0.34	0.11	0.24	0.69	−1.30
ND	Neurotactin	Cluster-13084.1222	0.03	0.09	0.60	−5.40	−3.62
ND	Neurotactin	Cluster-12411.0	0.04	1.25	0.56	−4.85	2.69*
ND	Neurotactin	Cluster-12411.1	0.01	0.51	0.24	−5.17	1.62*
ND	umknown (I)	Cluster-11107.0	0.00	0.09	0.20	**-**	−1.40
ND	umknown (I)	Cluster-8984.0	0.00	0.17	0.14	**-**	0.30
ND	umknown (I)	Cluster-16360.0	0.05	0.10	0.11	−1.27	−0.21

Note: * indicated that the expression level of one gene in guts (G) or antennae (A) was significantly higher than that in heads (H), with log2(fold) > 1 and P_adj<0.05. The heatmap scale was based on log10 (FPKM+1) values:Min = 0.0 Max = 2.93.

A total of 14 and 7 CCEs were significantly enriched in guts and antennae compared to heads (Table 6). Amongst the 32 HPP class genes, 14 exhibited higher expression levels in guts, whereas 5 were more expressed in antennae than heads (Table 6). Amongst the 15 ND class genes, almost all of them exhibited higher expression levels in heads than guts or antennae, except for two genes that were more expressed in antennae than heads (Table 6).

### 3.4 Identification and tissue-specific expression analysis of GSTs

**Fig. 5:**
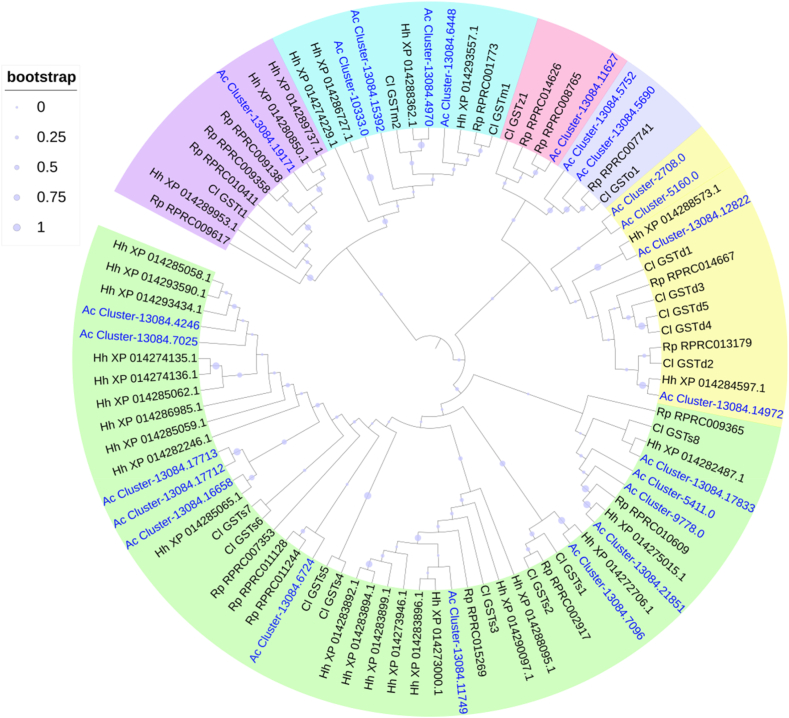
Phylogenetic tree of the GST genes of *A. custos* (Ac), *Halyomorpha halys* (Hh) [26], *Rhodnius prolixus* (Rp) [27,32] and *Cyrtorhinus lividipennis* (Cl) [15], constructed by the Maximum likelihood method with the JTT model. Expasion of the Sigma class in bugs were shown in the tree.

Insect GSTs can be grouped into seven classes named Delta, Epsilon, Omega, Sigma, Theta, Zeta, and microsomal [37]. In *A. custos*, 23 putative GST genes or gene fragments were found (Table S3; Table 7, Table 8). A phylogenetic analysis of GSTs showed that *A. custos* GSTs belong to six classes: delta (2 members), omega (2), sigma (12), theta (2), zeta (1) and microsomal (4) (Fig. 5). No epsilon GST gene was found in *A. custos*. The number of GST genes of different classes found in *A. custos* mirrors that reported in *O. laevigatus* (Table 7). Particularly, the sigma class was the largest group in *A. custos* and other bug species (Table 7).

**Table 7:** Numbers of GST genes annotated in *A. custos* (in this study), *Orius laevigatus* [31], *Cyrtorhinus lividipennis* [15], *Rhodnius prolixus* [27,32], *Triatoma infestans* [32], and *Halyomorpha halys* [26].

Class	*Arma custos*	*Orius laevigatus*	*Cyrtorhinus lividipennis*	*Rhodnius prolixus*	*Triatoma infestans*	*Halyomorpha halys*
Delta	2	1	5	1	1	2
Epsilon	0	0	0	0	0	0
Omega	2	2	1	1	0	3
Sigma	12	16	8	7	9	19
Theta	2	1	1	4	2	3
Zeta	1	1	1	1	0	1
Microsomal	4	3	2	1	2	5
Total	23	24	18	15	14	33

**Table 8:** FPKM values of GST genes in different tissues/parts of *A. custos*.

Class	Gene ID	Log10(FPKM+1)			Log2(Fold)	
		G	A	H	G/H	A/H
Sigma	Cluster-13084.11749	3.12	2.59	2.86	0.84	−0.90
Sigma	Cluster-13084.7025	2.17	1.05	1.14	3.53*	−0.33
Sigma	Cluster-13084.7096	1.70	0.64	1.03	2.35*	−1.54
Sigma	Cluster-13084.17713	1.62	0.00	0.07	7.90*	–
Sigma	Cluster-13084.16658	1.60	0.01	0.02	9.92*	−1.00
Sigma	Cluster-13084.10016	1.47	0.83	1.23	0.86	−1.47
Sigma	Cluster-13084.4246	0.08	0.41	1.29	−6.52	−3.56
Sigma	Cluster-13084.17712	0.86	0.00	0.00	**∞**	–
Sigma	Cluster-13084.21851	0.00	0.04	0.78	**-**	−5.81
Sigma	Cluster-9778.0	0.00	0.08	0.34	**-**	−2.56
Sigma	Cluster-5411.0	0.30	0.00	0.00	**∞**	–
Sigma	Cluster-13084.9790	0.25	0.23	0.24	0.10	−0.04
Delta	Cluster-13084.12822	2.95	2.06	2.52	1.43*	−1.54
Delta	Cluster-13084.14972	0.00	1.01	0.12	**-**	4.90*
Omega	Cluster-13084.5752	1.81	0.81	1.20	2.10*	−1.44
Omega	Cluster-13084.5690	1.15	0.74	1.13	0.06	−1.49
Theta	Cluster-13084.19171	0.77	0.50	0.96	−0.73	−1.91
Theta	Cluster-13084.17833	0.91	0.47	0.74	0.66	−1.23
Zeta	Cluster-13084.11627	1.03	0.86	1.05	−0.09	−0.73
Microsomal	Cluster-13084.6448	2.15	1.20	1.91	0.82	−2.42
Microsomal	Cluster-13084.4970	1.41	0.94	1.58	−0.57	−2.26
Microsomal	Cluster-10333.0	0.00	1.42	0.00	**-**	**∞**
Microsomal	Cluster-13084.15392	0.18	0.29	0.42	−1.69	−0.83

Note: * indicated that the expression level of one gene in guts (G) or antennae (A) was significantly higher than that in heads (H), with log2(fold) > 1 and P_adj<0.05. **∞** indicated that the expression level of one gene in heads was not detected. The heatmap scale was based on log10 (FPKM+1) values:Min = 0.0 Max = 3.12.

Compared to heads, 8 out of 23 GSTs showed higher expression levels in guts, including half (6/12) of GST sigma class genes (Table 8). Only two GSTs were significantly more expressed in antennae than heads (Table 8).

## 4 Discussion

In the present study, 91 CYPs, 50 CCEs and 23 GSTs were identified in a transcriptome analysis of different tissues or parts from fifth-instar nymph of *A. custos*. Notably, almost all of these genes of *A. custos* were more identical to their orthologues of *H. halys* in comparsion with those of *C. lividipennis*, *R. prolixus* and other bugs, supporting that predatory stink bugs were evolved from the phytophagous stink bugs [38,39]. The number of detoxification famliy gene members varying across insect species might be associated with the functional differentiation during insect evolution and related to the process of adaptation to the environment [40]. After comparing the number of these three famliy gene sequences in *A. custos* with those of the corresponding genes in other several Heteroptera species, we found a similar pattern of the member numbers of CYPs, CYP3, CYP4 and the HPP CCE class across Heteroptera species. *A. custos* contained more detoxification clan or class genes than other predatory bugs, *O. laevigatus* and *C. lividipennis*, but fewer than the herbivory stink bug *H. halys* and the hematophagous bug *R. prolixus*. The herbivory stink bug *H. halys* embracing the more expanded and diverse set of detoxification genes might due to its extreme generalist behavior [26,41]. Although *A. custos*, *O. laevigatus* and *C. lividipennis* are zoophytophagous that are carnivores and sometimes feed on plants, *A. custos* might need more detoxification genes to adapt more habitats and feed on more prey species [42]. These results indicate that CYP3, CYP4 and HPP CCEs relatively expanded in *A. custos* might be importance detoxification gene groups responding to more complex environment.

Our results also showed the different tissue-specific expression patterns of CYPs, CCEs and GSTs in *A. custos*, which provide useful information for the identification of candidated degrading genes responding to xenobiotics and odors. The insect gut serves as a major digestion organ, involved in digestion and detoxification of food and xenobiotics. A total of 37 CYPs, 14 CCEs and 8 GSTs were enriched in guts of *A. custos*, suggesting that they were proposed to have important roles on xenobiotic detoxification. In addition, 6 CYPs, 5 CCEs and 2 GSTs were up-regulated in *A. custos* antennae, indicating that these antenna-enriched metabolizing genes might have the role on ordorant degradation.

CYPs can readily catalyze the detoxification of chemicals by hydroxylation, epoxidation, dealkylations and a great variety of other monooxygenase reactions [43]. Our finding that most of CYP3 genes (37/48) were mainly expressed in guts of *A. custos* nymphs, showing that CYP3 clan members were the major detoxification gene group in the predatory stink bugs. Expression levels of CYP4 clan members were quite diverse among different tissues of *A. custos* nymphs. Seven gut-specific CYP4 genes of *A. custos* might be related to xenobiotic detoxification, whereas two CYP4 genes enriched in *A. custos* antennae might be ODEs.

The well-studied type of ODEs are P450s. The antennal enriched CYPs not only degrade plant volatiles and insecticides but also inactivate the pheromones in olfactory organs [13]. An antennal specific enzyme *Dendroctonus ponderosae* CYP345E2 that was the first olfactory CYP functionally characterized *in vitro* in an insect may catalyze the oxidation of many volatile monoterpenes for odorant clearance [44]. An antennal CYP gene *Spodoptera litura* CYP4L4 played a role in the recognition sex pheromones [45]. The identification of ten antennal specific CYPs of *A. custos* in the present study will be informative in the future studies about the chemical ecology of the predatory stink bugs.

Generally, insect CYPs in the CYP2 and mito clans have been regarded as conserved players in cell signaling and developmental processes, which is consistent with our observation that they were mainly expressed in heads of *A. custos* nymphs. The CYP2 and CYPmito clans contain several halloween genes for ecdysone and 20-hydroxyecdysone (20-E) synthesis (CYP302A1, CYP306A1, CYP307B1, CYP314A1 and CYP315A1), CYP18 for 20-E turnover, and CYP15 for juvenile hormone synthesis, CYP301 for cuticle formation, CYP303 gene for the structure and function of sensory organs, and CYP305 for unknown function [26]. But a recent study confirmed that some of them are also capable of metabolizing xenobiotics [46]. The potential roles of CYP2 and mito clan P450s of predaroty bugs in insect chemical defense remain unclear.

Recent studies suggested that DD CCE class is absent in hematophagous bugs, predatory *O. laevigatus*, and herbivory Pentatomidae bugs [31]. Our results revealed that DD CCEs are also absent in the predatory Pentatomidae bug *A. custos*, suggesting the lose of DD CCEs during the evolution of Heteroptera. In *A. custos*, most of known ND CCE class genes exhibited higher expression levels in heads, which suggests a potential function in neural development and signal transduction for these genes. Almost half of HPP CCE clade members (14/32) showed higher expression levels in guts of *A. custos*, suggesting that these HPP CCE genes might be involved in detoxification. What's more, five HPP CCE up-regulated in antennae might be ODEs.

CCEs is also one of the most well documented ODEs [47]. The first CCE enzyme related to olfaction in insects was an antennae-specific esterase (ApolPDE) from Giant silk moth, *Antheraea polyphemus* [48]. Several more antennal CCEs involved in odorant degradation in insects have been identified subsequently, such as esterase 6 (EST-6) in *Drosophila melanogaster* [49,50], SlitCXE7 and SlitCXE10 in *Spodoptera littoralis* [51,52], SexiCXE4 and SexiCXE14 in *S. exigua* [53,54]. The expression in a specific tissue could shed lights on the function of a CCE. Several antennal-specific CCEs in *A. custos* might be involved in the hydrolysis of host plant volatiles, prey odorants and pheromone components, but further studies are needed.

GST enzymes play a fundamental role in the xenobiotic detoxification, hormone biosynthesis and protection against oxidative stress [[5][5], [5][6], [5][7]]. The expansion of the sigma class was found in *A. custos*, which is consistent with other studies reported in several hemipteran species [31]. In *A. custos*, half of the sigma class (6/12) showed more expression levels in guts, supporting the link of a lineage specific expansion of the GST sigma class in Hemiptera to xenobiotic metabolism. In other insect species, the sigma class was reported to link with insecticide detoxification [[5][8], [5][9], [60], [6][1], [6][2], [6][3]].

GSTs that are crucial in odorant degradation as the olfactory genes of insects generally show preferential expression in the antennae, for example, GST-msolf1 of *M. sexta* [64], GST-pxcs1 of *P. xuthus* [65], GmolGSTD1 of *Grapholita molesta* [66] and SzeaGSTd1 of *Sitophilus zeamais* [67]. In *A. custos*, only two antennae-specific GSTs were identified, whose functions related to odorant degradation needed be further investigated.

Predatory stink bugs are zoophytophagous generalists of various insect pests in agriculture and forest plantations [68]. *A. custos* feeds on many species of pest insects and perfers to live in elm, poplar, cotton, soybean and other plants [2]. Stink bugs (Pentatomidae) produce unpleasant scent odors that function as alarm and defense signals against natural enemies [69]. Therefore the predatory stink bugs depend on their antennae to perceive a diversity of airborne chemical cues, including prey odorants, plant volatiles, sex pheromones and scents from stink bugs, to find palatable preys, preferable host plants and conspecific partner, and to avoid interspecific competition and natural enemies. These insects rapidly respond to stimuli in their environment, mainly relying on the termination of odorant signals from the olfactory sensilla following stimulation by ODEs [13]. A number of candidate *A. custos* ODEs that were identified in the present study will be informative in the future studies about the elucidation of their olfactory system and the improvement of their bio-control efficiency.

## 5 Conclusions

In the present study, the comprehensive analysis of xenobiotics detoxification and odor degradation genes in the predatory stink bug were conducted. A total of 91 CYPs, 50 CCEs and 23 GSTs were identified based on RNA-seq transcriptomic analysis. Tissue-specific expression analysis showed that most of gut-enriched detoxification enzyme genes belonged to CYP3 and CYP4 clans, HPP CCE and GST Sigma classes, whereas antennae-specific odorant degrading genes were contained in CYP4 clan and CCE HPP class. A further in-depth study examined these xenobiotics detoxification and odor degradation genes will provide basic information for studying the improvement of the bio-control efficiency of the predatory stink bugs.

## Author contribution statement

Wenhong Li: Conceived and designed the experiments; Performed the experiments; Wrote the paper.

Jingmiao Zou; Xiang Yang: Performed the experiments.

Mingwei Yang: Performed the experiments; Contributed reagents, materials, analysis tools or data.

Po Jiang; Xinyi Wang: Contributed reagents, materials, analysis tools or data.

Chunyang Huang: Conceived and designed the experiments; Analyzed and interpreted the data; Contributed reagents, materials, analysis tools or data; Wrote the paper.

Yueping He: Conceived and designed the experiments; Analyzed and interpreted the data; Wrote the paper.

## Data availability statement

Data associated with this study has been deposited at NCBI SRA with BioProject accession number of PRJNA878928.

## Additional information

Supplementary content related to this article has been published online at [URL].

## Declaration of competing interest

The authors declare that they have no known competing financial interests or personal relationships that could have appeared to influence the work reported in this paper.

## Appendix A Supplementary data

Fig. S1 Verification of primer specificity using PCR and melting curves Fig. S1

Table S1 Primers used in this study Table S1

Fig. S2 Similarity distribution (A) and species distribution (B) of unigenes that hit in the NCBI NR protein database Fig. S2

Fig. S3 Correlation between quantitative real-time PCR (qRT-PCR) dCT data and RNA-seq FPKM data Fig. S3

Table S2 Transcriptomic analysis information Table S2

Table S3 Predicted protein sequences of CYP, CCE and GST genes detected in *A. custos*. Table S3
