# Supplementary material for: Identification of metabolizing enzyme genes associated with xenobiotics and odorants in the predatory stink bug Arma custos based on transcriptome analysis
Source: Heliyon. 2023 Jul 27;9(8):e18657. doi: 10.1016/j.heliyon.2023.e18657 (PMC10412767; doi:10.1016/j.heliyon.2023.e18657)
Supplement: Fig. S1 — Verification of primer specificity using PCR and melting curves [file mmc1.docx]

Figure S1 Verification of primer specificity using PCR and melting curves
