# Supplementary material for: Identification of metabolizing enzyme genes associated with xenobiotics and odorants in the predatory stink bug Arma custos based on transcriptome analysis
Source: Heliyon. 2023 Jul 27;9(8):e18657. doi: 10.1016/j.heliyon.2023.e18657 (PMC10412767; doi:10.1016/j.heliyon.2023.e18657)
Supplement: Table S1 — Primers used in this study [file mmc2.docx]

**Table S1 Primers used in this study**

| **Primer name** | **Primer sequence** | **Amplicon Length（bp）** |
| --- | --- | --- |
| Cluster-13084.11702-F | CTTCTGGCAGCAACGGTT | 123 |
| Cluster-13084.11702-R | GAAGTTGCCGACTAGAGG |  |
| Cluster-13084.11749-F | GTCGCTGTTTCTGAATAC | 128 |
| Cluster-13084.11749-R | GGTCTTTTGTTGAGCCAT |  |
| Cluster-13084.16634-F | CCAGTGAGCGACCATTTC | 103 |
| Cluster-13084.16634-R | CCCAAAACTTCTCCTCGC |  |
| Cluster-13084.12300-F | GAGGCTGAAGGAAATGGG | 106 |
| Cluster-13084.12300-R | CCAATCCTCTGGGGCTTT |  |
| Cluster-13084.10808-F | GTTCTTGGTTTTCTCAGC | 98 |
| Cluster-13084.10808-R | GCTATGTTCTGCTTTACC |  |
| Cluster-13084.11247-F | GGTACAGCCATCCTCATT | 120 |
| Cluster-13084.11247-R | AACGATTGGAAGAGCTGG |  |
| Cluster-13084.10019-F | GCACGAAGACGAAGACAC | 89 |
| Cluster-13084.10019-R | CTGTAACCTGACGTGAAG |  |
| Cluster-13084.10017-F | GATGGACATCTTCTCCCT | 83 |
| Cluster-13084.10017-R | CTATGTACGGTTCGTTCC |  |
| Cluster-13084.17142-F | CCTTTGCTTGATGGCCTG | 96 |
| Cluster-13084.17142-R | CCGACGATCGGTGATGTA |  |
| Cluster-13084.16699-F | CTTGATTCCTGCTGGTAG | 140 |
| Cluster-13084.16699-R | CATAGGAATAAGGGTGTC |  |
| Cluster-13084.7214-F | GAGGCACATCAGAATGAA | 116 |
| Cluster-13084.7214-R | CACCTATTCCAAAAGGAG |  |
| Cluster-13084.6040-F | GGGCATACTGATGAGATC | 128 |
| Cluster-13084.6040-R | GCTTTCCCATGCTTAGCA |  |
| Cluster-13084.13485-F | TATCCCATATGCGAAACC | 72 |
| Cluster-13084.13485-R | CCTAACCATGGGCTTGCT |  |
| Cluster-13084.12822-F | GCACCTTACGATGAAGAG | 118 |
| Cluster-13084.12822-R | GAGCAAGATCAGCCAAAG |  |
| Cluster-13084.14972-F | CCTTCTCTCTCAGTGATT | 84 |
| Cluster-13084.14972-R | CTGCTCTTTACCCAGGAT |  |
| Cluster-10333.0-F | CTTCGCGGGTTACGCATT | 131 |
| Cluster-10333.0-R | CTACTCCTGCTGCTATTG |  |
| Cluster-13084.11009 (EF1A)-F | GTCATTCCAAGAGTTCCC | 128 |
| Cluster-13084.11009 (EF1A)-R | CGGCGGCCTTTGTTACTT |  |
