# Supplementary figures and images for: Identification of metabolizing enzyme genes associated with xenobiotics and odorants in the predatory stink bug Arma custos based on transcriptome analysis
Source: Heliyon. 2023 Jul 27;9(8):e18657. doi: 10.1016/j.heliyon.2023.e18657 (PMC10412767; doi:10.1016/j.heliyon.2023.e18657)

## Slide 1
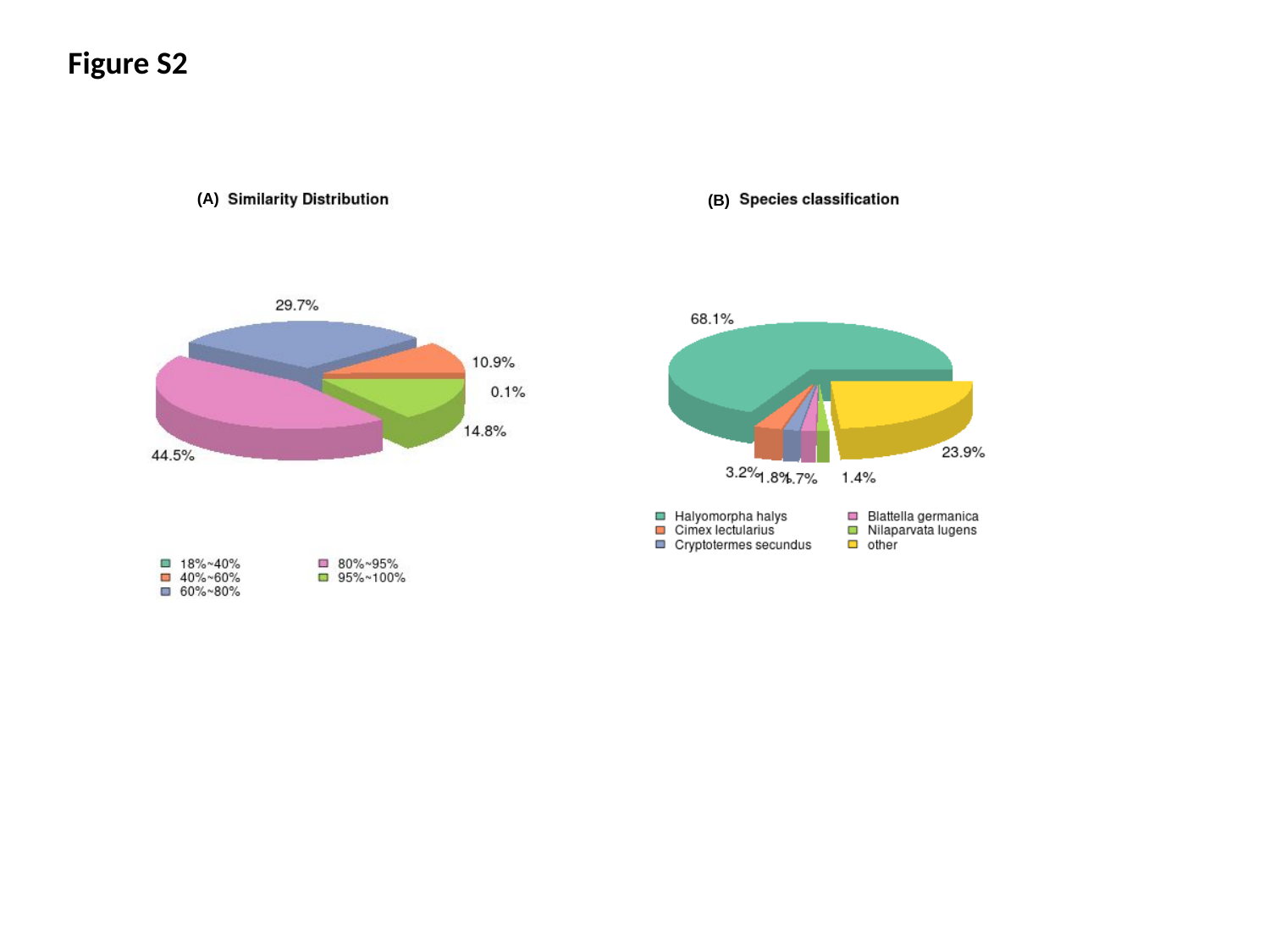

Figure S2
(A)
(B)

Supplement: Fig. S2 — Similarity distribution (A) and species distribution (B) of unigenes that hit in the NCBI NR protein database [file mmc3.pptx]

## Slide 1
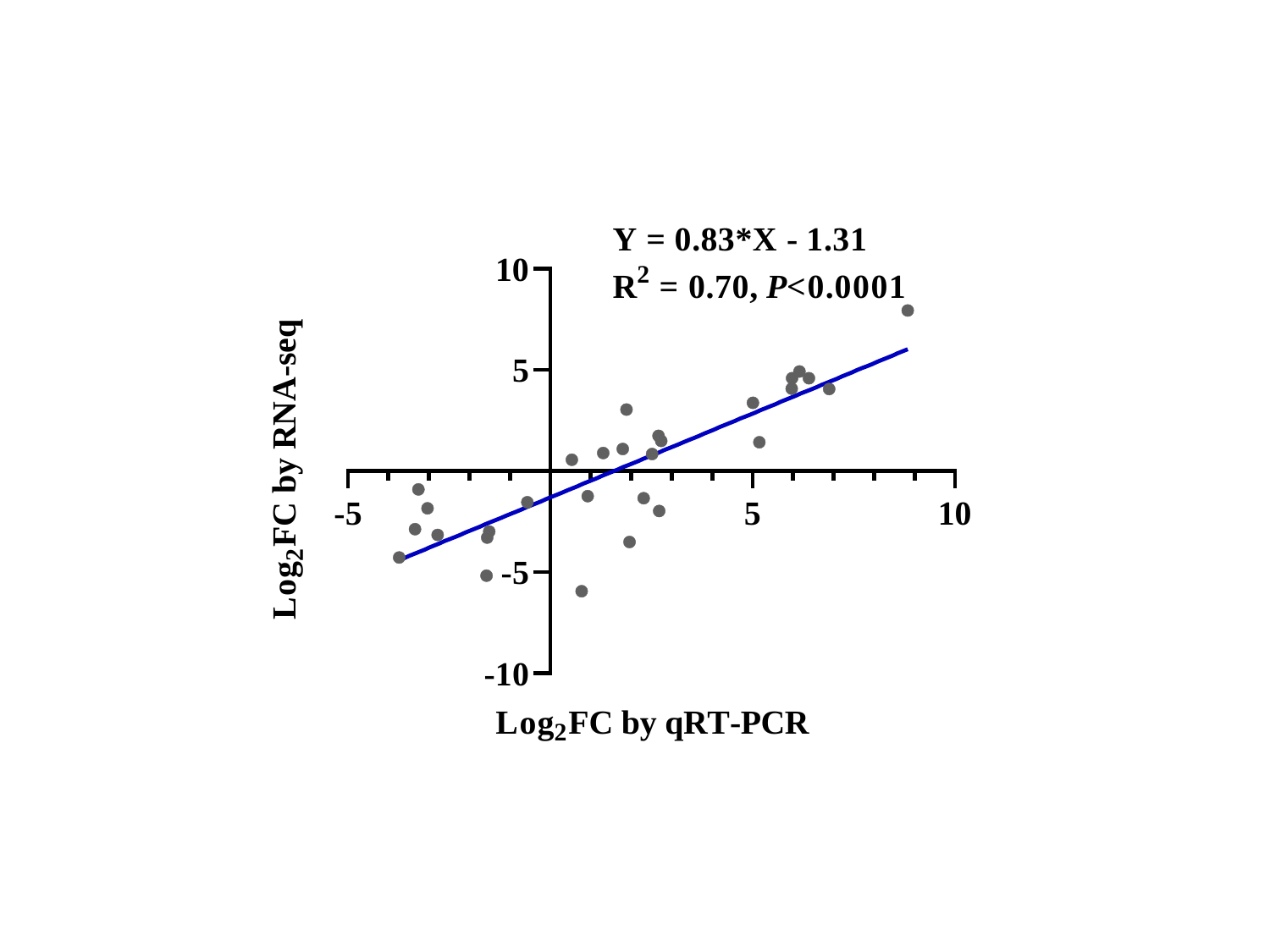

Supplement: Fig. S3 — Correlation between quantitative real-time PCR (qRT-PCR) dCT data and RNA-seq FPKM data [file mmc4.pptx]
